# DKC1 mutation causing different phenotypes in a family with X-linked Hoyeraal-Hreidarsson syndrome

**DOI:** 10.1186/1939-4551-8-S1-A209

**Published:** 2015-04-08

**Authors:** Karine Boufleur, Carlos Alberto Scrideli, Elvis Valera, Luiz Tone, Tom Vulliamy, L Karla Arruda, Persio Roxo Jr

**Affiliations:** 1 Ribeirao Preto Medical School, University of Sao Paulo, Brazil; 2 Clinical Hospital of Ribeirao Preto School of Medicine, Brazil; 3 Centre for Paediatrics, Blizard Institute, Barths and the London School of Medicine and Dentistry, UK; 4 Brazilian Association of Allergy and Immunology, Brazil

## Background

Hoyeraal–Hreidarsson syndrome (HHS) is a rare multisystem disorder characterized by intrauterine growth retardation, microcephaly, cerebellar hypoplasia, neurological deficits, aplastic anemia, and immunodeficiency. HHS is a severe variant of Dyskeratosis Congenita (DC) that displays clinical features overlapping DC, with T, B and NK immunodeficiency. Both genetic disorders presents deficiency in maintaining telomere integrity. Mutations in the *DKC1* gene are responsible for the X-linked form of the disease.

## Methods

A case report of a two-year-old male referred for investigation because of a family history of HHS.

## Results

The patient had no history of recurrent infections, diarrhea or vaccine reactions. Weight in percentile 3 and height in 10. He also had nail dystrophy, leukoplakia of the tongue and normal neurological development. Laboratory tests: lymphopenia and mild thrombocytopenia (biopsy not performed due to family refusal); normal immunoglobulins and IgG subclasses levels; pre-and post-immunization levels of IgG to pneumococcal polysaccharides measured by ELISA were low for all tested serotypes; low levels of NK cells and lymphocyte subpopulations CD4+,CD8+,CD19+, as well CD4+/CD8+ ratio. Magnetic resonance imaging (MRI) of the brain showed a mild enlargement of magnum cistern, without cerebellar hypoplasia. Endoscopy showed an oesophageal stenosis (attributed to HHS). Genetic analysis by Polymerase Chain Reaction showed a missence mutation of the *DKC1* exon 11 (Ala353Val). His older brother, who presented the same mutation, had a previously history of recurrent and severe infections since his first month of life with evident neurological delay, transfusion dependent bone marrow failure, cellular and humoral immunodeficiency and the complete clinical phenotype of HHS. Cerebellar hypoplasia was observed at brain's MRI.

## Conclusions

As identical mutations can lead to different manifestations it is likely that other homozygous or heterozygous genetic alterations not yet known might also contribute to HHS. Our familial cluster exemplifies how diverse the clinical phenotype can be between relatives carrying the same DKC1 point mutation. This clinical information may be important for counseling, as affected families may be very concerned about the potential clinical outcome of their offspring affected by this serious disease.

## Consent

Written informed consent was obtained from the patient for publication of this abstract and any accompanying images. A copy of the written consent is available for review by the Editor of this journal.

